# Arul M. Chinnaiyan, MD, PhD, and Charles L. Sawyers, MD, receive the 2026 Harrington Prize for Innovation in Medicine

**DOI:** 10.1172/JCI211106

**Published:** 2026-08-03

The American Society for Clinical Investigation (ASCI) honors Arul M. Chinnaiyan,MD, PhD, and Charles L. Sawyers, MD, with the 2026 ASCI/Harrington Prize for Innovation in Medicine. Drs. Chinnaiyan and Sawyers are recognized for their discoveries defining the molecular drivers of prostate cancer and pioneering precision therapies that have reshaped the diagnosis and treatment of prostate cancer worldwide. Dr. Chinnaiyan is the S.P. Hicks Endowed Professor of Pathology and Section Head, Michigan Center for Translational Pathology, at the University of Michigan, and a Howard Hughes Medical Institute (HHMI) Investigator. He was elected to the ASCI in 2006. Dr. Sawyers is the Marie-Josée and Henry R. Kravis Chair in Human Oncology and Pathogenesis and Chair, Human Oncology and Pathogenesis Program, at Memorial Sloan Kettering Cancer Center, as well as an HHMI Investigator. He was elected in 1999. ASCI Secretary-Treasurer Goutham Narla, Louis Newburgh Research Professor of Internal Medicine at the University of Michigan School of Medicine, interviewed the award recipients in June 2026.

Goutham Narla: Dr. Chinnaiyan and Dr. Sawyers have inspired many of us to pursue the pathways and careers that we’ve chosen, and it’s an honor to be able to speak to them. Dr. Sawyers, you’ve made seminal discoveries that have transformed care first in patients with chronic myeloid leukemia (CML) and subsequently [those with] prostate cancer. Can you tell us a little bit about that journey and how you’re able to pivot and be able to make discoveries in two different fields at that level?

Charles L. Sawyers: I wish I could say that it was a planned pivot, but things just happen. When I went to medical school, I didn’t know I wanted to be a scientist initially, and I got bit by the bug when I was assigned my roommates as a first-year medical student. I was in a quad with a bunch of MD-PhD students, one of whom [was] Bill Nelson, who’s now the Cancer Center Director at Hopkins. About halfway through the first year, I [thought], What am I doing? I should probably be doing *this*. I didn’t switch into the MD-PhD program, but I did become passionate about science. The reason I’m telling you that is because I became fascinated with leukemia as not only a topic in medical school, and the biology and the translocations and so forth; it just seemed like the disease where the chance to molecularly understand driver alterations, as we call them now, was possible.

I intentionally sought out Owen Witte’s laboratory at UCLA after his was the first group to clone the BCR-ABL translocation in CML. I got off the clinical training track and became a postdoc in his lab for five years. That’s how I got interested in kinase signaling. Then Gleevec appeared, and it was an incredible time, as I think everyone knows. To get back to how did I pivot to prostate cancer: the work in Gleevec was going really well, and one of the main problems that presented itself was acquired resistance. We were able to decipher that problem by sequencing patients who were responding and then during remission and relapse, and we discovered kinase domain mutations as the cause of resistance. That led to next-generation drugs. The problem is not completely solved now, but patients with CML have a normal lifespan at this point. I was in my mid- to late 40s and [thought], I’ve got to work on something else. It’s not exactly as clean a pivot as that, but I wondered, What is another drug resistance problem? Because I felt like I had a way of thinking about it that seems obvious now but wasn’t so prevalent. So I started pivoting to prostate cancer to ask the question about hormone resistance.

GN: Dr. Chinnaiyan, to build upon that: Dr. Sawyers and others identified key drivers, fusion proteins that drive hematological malignancies, and you went on to identify some key ones in prostate cancer. Can you take us through that journey? What allowed you to think outside the box that the same paradigms that apply to hematological malignancies might also apply to certain solid tumors as well?

Arul M. Chinnaiyan: My training path was somewhat unusual. I completed essentially all of my scientific and medical training at one institution, the University of Michigan. I started here as an undergrad and then pretty much never left. Michigan provided an extraordinary environment for both clinical training and scientific exploration. My research trajectory really took off during my MD-PhD program in the Medical Scientist Training Program. I did my graduate work in the laboratory of Vishva Dixit, who was then a professor of pathology at Michigan before he moved on to Genentech-Roche. In Vishva’s lab, I was fortunate to be part of an early effort to define the components of programmed cell death, or apoptosis. This was a very exciting time for me scientifically. I had the opportunity to clone and characterize a number of key components of the cell death pathway, including FADD and FLICE, better known as caspase-8 nowadays, as well as the death receptors DR3 and DR4. That was, at least as a formative thing for me, certainly a magical time in the research laboratory.

After completing my PhD with Vishva, I returned to clinical training and ultimately chose pathology for my residency. At the time, the chair of pathology was Peter Ward, who also served on my PhD committee. He offered me an opportunity to start my own laboratory at Michigan while completing my residency. I thought that this was an extraordinary opportunity and one of the major reasons that I continued at Michigan. When I began my independent laboratory, I became very interested in emerging genomic technologies, bioinformatics approaches, and this was the pre–next-generation sequencing era. This is when cDNA microarrays were all the rage, and it was really the most powerful tool at the time to study cancer biology at scale. I was one of the first investigators to establish cDNA microarray technology at the University of Michigan, techniques that I learned from Pat Brown’s lab at Stanford. With those tools, my lab and many others began to profile human cancers to identify patterns of gene expression to reveal new biology.

Around that time, my lab developed a resource called Oncomine, a cancer microarray database analysis platform. Oncomine allowed us to systematically compare gene expression patterns across many tumor types and to look for genes that are highly abnormal or had an outlier expression in a subset of patients. I think that led me naturally to work on prostate cancer, primarily because Michigan had a strong prostate cancer program, including well-annotated tumor biorepositories. We had an NCI SPORE program, which made it an ideal setting to apply genomic technologies. We began to profile prostate cancer samples using that microarray technology. By combining that data with this outlier analysis approach we developed through Oncomine, we identified that ETS transcription factors showed this striking outlier expression in subsets of prostate cancers. That observation prompted us to ask why these ETS genes were so highly expressed in certain tumors. Scott Tomlins, who was a talented MD-PhD student in my laboratory at the time, led the follow-up work using 5′ RACE technology to characterize the upstream sequences of these transcripts. That work led to the serendipitous discovery of recurrent TMPRSS2-ETS gene fusions in prostate cancer, and particularly TMPRSS2-ERG, which is found in a very large fraction of patients.

At the time, it was a surprising and paradigm-shifting observation. Before then, recurrent gene fusions were largely thought of as defining lesions in hematologic malignancies, such as CML, a disease so beautifully studied by Charles Sawyers and others. The idea that common epithelial tumors like prostate cancer could be driven by recurrent gene fusions was not really appreciated. In general, my path to studying these recurrent genetic defects in prostate cancer came from the convergence of a few things: training in molecular mechanisms of disease, clinical pathology, early adoption of genomic technologies, access to exceptional prostate cancer specimens at Michigan, and a willingness to let unbiased data point us toward unexpected biology.

GN: One of the things that’s been truly inspirational for all of us about both of your careers is your strong focus on basic discovery science, but with an eye toward translation. Perhaps you could speak about the importance of basic mechanistic science and its role in drug development, especially in the current environment. In addition, how do we couple that discovery to true translation to our patients and the role of, for example, venture capital and industry in that process?

CLS: Obviously, it’s a really important question because of today’s climate in particular. There’s no doubt that everything that I’ve done translationally was built on a foundation of basic discovery that in many cases went back 20, 30 years. For young physician-scientists, it’s important to not just read those papers and act on them. It’s to get the experience of doing basic science yourself, because I think you need to understand what’s under the hood. You can’t just read the abstracts. I go back to the story Arul just told. I was a heme malignancy guy because that’s where the translocations were. And why were they there? We thought it was something about heme cells. It was because we could grow those cells in culture and see them and do metaphases. But to Arul’s credit, that wasn’t possible with prostate cancer or other cancers, but he used another way of thinking about it. Arul, the way you told the story, which is the way I understand it when I first met you, you didn’t think it was a translocation. You just used clever observation to ask, Gee whiz, why is this thing so upregulated? So maybe that’s the best example. If the technologies to do that kind of gene expression array analysis hadn’t been invented through a lot of basic, even engineering technologies, we wouldn’t perhaps know what we know today.

AMC: I could follow up a bit in terms of a team project that I had an opportunity to co-lead with Dr. Sawyers, the Stand Up To Cancer–Prostate Cancer Foundation Dream Team, which began with basic science that then translated into some intriguing clinical observations that have had patient impact, but really wouldn’t have been possible without some of the foundational work in terms of profiling metastatic castration-resistant prostate cancer. I’ve been a longtime admirer of Charles’s work. His contributions in understanding CML, a disease driven by a recurrent gene fusion, were foundational [for] understanding the resistance mechanisms, [as was] his particular pivot into prostate cancer, which is also a gene fusion–driven cancer. There’s been a natural connection between our work, primarily because the gene fusions we identified in prostate cancer, particularly TMPRSS2-ERG, are androgen regulated. So in a sense, androgen receptor — which of course testosterone, the male hormone, utilizes — is directly linked to the expression of these recurrent gene fusions as important drivers of prostate cancer; and when you therapeutically impact androgen signaling, you’re essentially shutting off the gene fusion.

Our efforts in terms of the precision oncology space converged with Charles’s efforts, especially in this area; [this] was initiated through our first clinical sequencing program for advanced cancer called MI-ONCOSEQ that we developed here at the University of Michigan. This is an effort to, in the next generation after microarray technology, bring on next-generation sequencing and [see] how that can be incorporated into precision medicine workflows, starting with tumor sequencing, integrating germline sequencing, and then returning those results to patients. One of the exciting team projects that I’ve had the fortune to work with Charles on has been this Dream Team. This allowed us to take these precision medicine approaches at scale across multiple institutions. The Dream Team itself was one of the first major multi-institutional precision oncology efforts in prostate cancer. It brought together leading groups across the United States and the United Kingdom, linking clinical expertise, genomic sequencing, pathology, bioinformatics, and translational research in a coordinated way. But one of the most important aspects of the project was it focused on patients with metastatic castration-resistant prostate cancer. At the time, very little was known about this patient population because these samples were harder to get at than, for example, clinically localized prostate cancer samples. What we found through this project was that advanced prostate cancer wasn’t a single molecular entity, but rather a disease with distinct genomic subsets that could have therapeutic implications. One of the most impactful findings from that effort was that a substantial fraction of the patients, about a quarter of them, had alterations in DNA repair pathways. This included genes such as BRCA2, BRCA1, mismatch repair genes, CDK12, and others. Interestingly enough, a good fraction of those alterations were occurring in the germline. So they had implications not only for the patient, but the patient’s family in general. But in terms of [clinical translation of a foundational observation], this is where the Stand Up To–Cancer–Prostate Cancer Foundation team effort had the impact, because it laid the foundation for PARP inhibitors in metastatic castration-resistant prostate cancer, in that patients with [alterations in] BRCA2, BRCA1, and related DNA repair genes could potentially benefit from PARP inhibitors. More broadly, the Dream Team showed the power of collaboration at scale, not only moving foundational observations to translational impact, but allowing us to work together in a major collaborative effort.

CLS: I’d add that there was no hypothesis behind that project. It was a technology; it was a moment in time when technology and then the opportunity to use the resources of Stand Up To Cancer and the Prostate Cancer Foundation philanthropy to do something that needed to be done. The Cancer Genome Atlas had sequenced 500 localized prostate cancers. So we said, Let’s sequence 500 castration-resistant prostate cancers. But we really didn’t know what we would find. The point I’m trying to make is, in a case like that, sometimes big science is the right solution, and then small projects or big singles will come out. The BRCA observation is unbelievably important clinically, and eventually we would have found it, but it’s really great. Another thing in big science projects like that that Arul and I did is we built a community of people; you have to get a buy-in into data sharing and credit and all that kind of stuff. So we spent a lot of time thinking about that and giving younger members of the team the leads on different papers and different spin-out stories, presentations at meetings, and so forth. So there’s a whole generation of what were the assistant professors and senior postdocs from that work that are now leaders in the prostate cancer field.

GN: Given that at the time we trained, some of our science was perhaps more limited by the available technologies, and now we can do next-generation sequencing at scale and whole genome sequencing, and with generative AI and such: How do we integrate the state-of-the-art technology that we have available to us today into science while still staying true to our purpose and understanding that fundamental mechanism–based science is still critical for translation?

CLW: I don’t have a great answer for the AI part, because I feel like I’m almost too old to learn that. But I do have a Claude account. I serve as an advisor to a biotech company that has as its goal AI drug design. It’s not clear that undruggable targets suddenly are solvable with this technology. Because as we all know, the power of this is pattern recognition that humans can’t see. If there’s no knowledge about a target that’s undruggable, I don’t see how it can just be dreamed up. But we’ll see. The point I would make is that you need to partner with people who have technological savvy. Science is more collaborative than it ever was by necessity, because I don’t think anyone could be great at all of these different tools, but they all are critical to be brought to bear on the questions that we’re interested in.

AMC: I think AI and enhanced-AI approaches are enabling and allowing others that are playing at a certain level of expertise to bring in diverse expertise. And those are the approaches that we’re exploring. But, again, it’s early days. It’s exquisitely important to focus on understanding biology and mechanisms of disease. I think there’s only so much that AI is going to really help in that context. But I see it as an enabling tool more than anything else.

GN: You’re both products of incredible mentors, Dr. Dixit, Dr. Witte, and many others. For the next generation of trainees, what are the most important lessons you’ve learned?

CLS: In my case, it was going to a lab on the cutting edge of something exciting that you really want to be part of. And you’ve got to show up in a major way. That’s for anything, I guess. I learned a lot from Owen. I also learned a lot from the other people in the lab. I think it’s important to join a lab that has a critical mass. Giant labs are probably a mistake — but maybe not. But I think really small labs — I worry that it’s hard to get enough experience. You learn just as much from your failed experiments and you learn how to get around those, not only from your mentor, but from the people around you at the bench. And if it’s not in your lab, it’s in the lab next door. Talk to people all the time during your training about your work, as long as they’ll listen to you. You have to learn your elevator pitch to get them excited and also be really interested in their work, because it’s incredible how much you learn about your own project from seeing how someone else solves a problem in their story that seems remotely related but may have a critical kernel of insight.

AMC: One lesson that I learned was that career paths don’t have to really be linear to be successful. I essentially did all of my training at the University of Michigan, which is unusual. But what mattered was not changing institutions for the sake of change. It was finding new scientific environments, new mentors, new technologies, and new problems that pushed me to keep growing. As you alluded to, Goutham, I would say that mentorship was absolutely critical. I was fortunate to have extraordinary mentors at different stages of my career. I mentioned Vishva and Peter Ward, many others who I think gave me scientific freedom, challenged me intellectually, and created opportunities for me. I try to pass that forward to my own trainees. I also learned the value of being open to unexpected directions, especially in the context of the discovery around the prostate cancer fusions. Many of the most important opportunities in science come from following the data, even when it takes you somewhere that you didn’t expect.

Then in terms of combining MD and PhD training, I would say that my clinical training in pathology also shaped the way that I think, seeing disease through the lens of patient samples, diagnosis, and clinical need. For our trainees, it’ll be important to stay connected to the human disease that you’re studying, whether you’re a physician-scientist, basic scientist, computational biologist, or a translational researcher. Also, choosing an important problem: again, techniques and approaches, AI approaches, all of this will change as the field evolves, but if you’re working on an important question that matters to you deeply — the biology and translating that in terms of medicine ultimately to patients — you’ll have a reason to keep on going. Last, being comfortable crossing boundaries, especially with the advent of AI approaches nowadays: some of the most exciting advances happen at this interface of disciplines in terms of, in my case for example, pathology and genomics, computation, clinical oncology, chemistry, and cancer biology. Trainees that can speak in more than one scientific language will be particularly well positioned in today’s age.

GN: I’d like to thank you both and congratulate you on receiving this Milestone Award.

AMC and CLS: Thank you so much.

*The interview has been edited for length and clarity*.

## Figures and Tables

**Figure 1:**
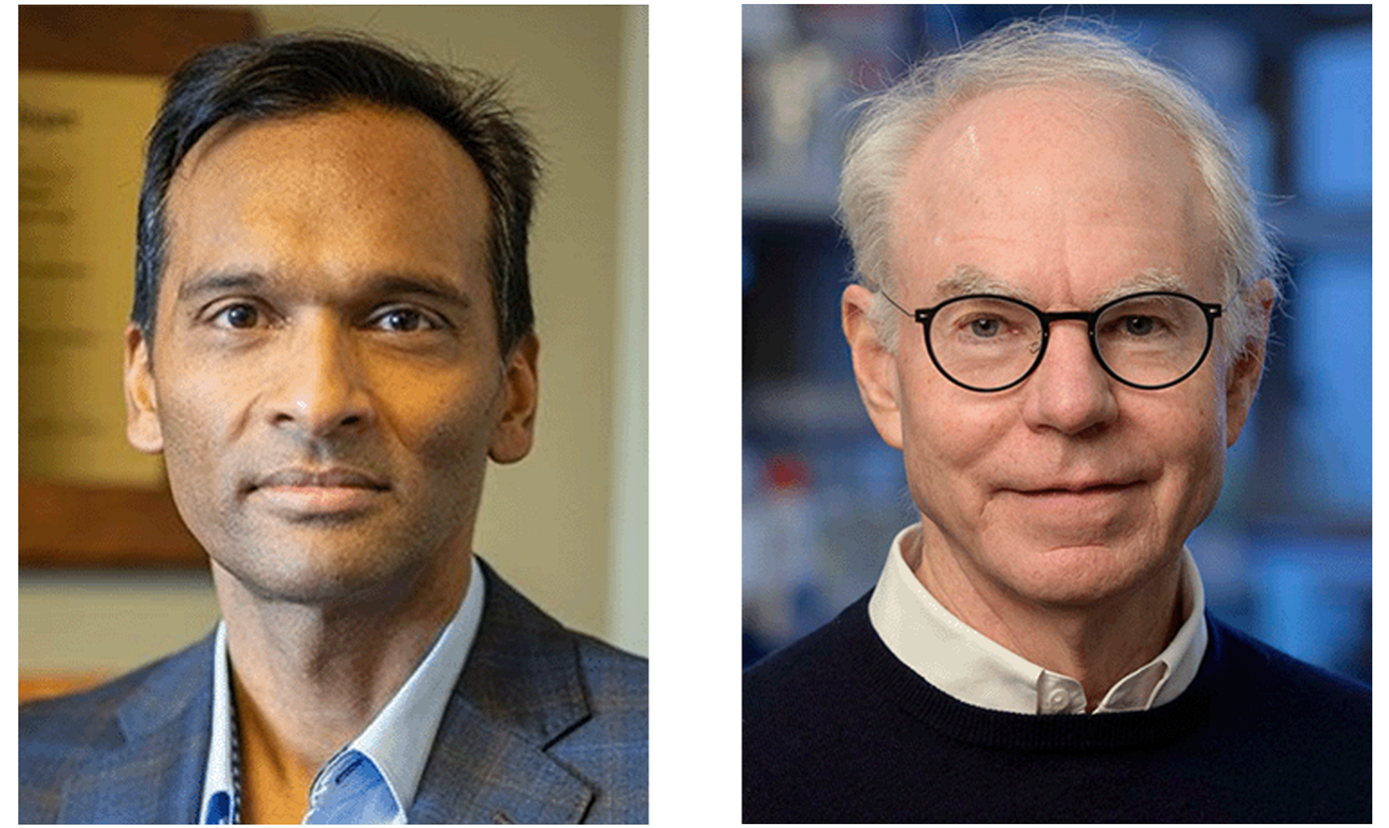
Arul M. Chinnaiyan (left) and Charles L. Sawyers (right) are the recipients of the 2026 Harrington Prize for Innovation in Medicine.

## Version 1 08/03/2026

Electronic publication

